# Interpretative problems due to the presence of chloromethcathinone isomers in the biological material from postmortem cases

**DOI:** 10.1093/jat/bkad070

**Published:** 2023-09-12

**Authors:** Agnieszka Romańczuk, Sebastian Rojek, Kamil Synowiec, Martyna Maciów-Głąb, Karol Kula

**Affiliations:** Department of Forensic Medicine, Faculty of Medicine, Jagiellonian University Medical College, Grzegórzecka 16 St., Kraków 31-531, Poland; Department of Forensic Medicine, Faculty of Medicine, Jagiellonian University Medical College, Grzegórzecka 16 St., Kraków 31-531, Poland; Department of Forensic Medicine, Faculty of Medicine, Jagiellonian University Medical College, Grzegórzecka 16 St., Kraków 31-531, Poland; Department of Forensic Medicine, Faculty of Medicine, Jagiellonian University Medical College, Grzegórzecka 16 St., Kraków 31-531, Poland; Department of Forensic Medicine, Faculty of Medicine, Jagiellonian University Medical College, Grzegórzecka 16 St., Kraków 31-531, Poland

## Abstract

While many new psychoactive substances often disappear from the drug market rather quickly, some, such as synthetic cathinones (SCs), still remain due to their popularity among users. The current knowledge of SC concentrations in blood samples is based mainly on the published case reports of intoxications or fatalities caused by SC intake. The aim of the present study was to present and interpret the obtained toxicological analysis results of these cases, in which it was possible to determine or detect the presence of one of the isomers of chloromethcathinone (CMC) along with its intake biomarker—dihydro-CMC. These cases include 27 deaths reported at the Department of Forensic Medicine in Kraków in 2016–2022. CMC constitutes a major toxicological opinion challenge, in terms of toxicological evaluation of poisonings. As presented in this paper, a significant problem is its stability in the biological material and practices in the reporting of the obtained data. It is therefore important to monitor potential intake biomarkers that may show greater stability in the biological material than the parent drug. In the case of CMC isomers, the good biomarker of intake is the dihydro-CMC metabolite, which was detected in the blood sample in every case presented, even with the absence of the parent substance. Interpretation of the results obtained for CMC in terms of assessing their toxicity and possible cause of death is difficult. However, it should be taken into account that in cases of new psychoactive substance poisoning, an in-depth risk assessment is mandatory and the opinion of the unpredictability of the effects is taken as a principle.

## Introduction

As the latest report from the European Monitoring Center for Drugs and Drug Addiction (EMCDDA) shows, new psychoactive substances (NPSs) are steadily appearing on the drug market, and with them the resulting risks. At the end of the year 2022, the EMCDDA monitored ∼930 NPSs, 164 of which were synthetic cathinones (SCs). This makes them the second largest category of NPSs monitored by the European Union’s Early Warning System, after synthetic cannabinoids (1).

While many NPSs often disappear from the drug market rather quickly, some are still on the market due to their popularity among users. SCs are popular due to their psychostimulant and hallucinogenic effects, which mimic the effects of cocaine, 3,4-methylenedioxymethamphetamine (MDMA) and amphetamine (2). SCs are a group of compounds that are a derivative of cathinone, which is a natural alkaloid found in the plant *Catha edulis* Forsk (khat). Structurally, they are β-keto analogs of amphetamine. The first SCs were synthesized in the late 1920s because of their potential therapeutic effects as antidepressants and appetite suppressants (3). Although several SCs have entered clinical trials for potential use as drugs, only bupropion is currently used as a smoking cessation aid, as well as in the treatment of depression. Studies have been conducted on other cathinone derivatives, but they have not been successful due to their serious side effects. Numerous cases of poisoning and deaths related to SC consumption have been reported. Their intake in high doses can cause symptoms including hypertension, hyperthermia, tachycardia and convulsions, which, in some cases, result in multiple organ failure and consequently death (2).

Chloromethcathinone (CMC), which is a chlorine derivative of methcathinone, exists in the form of three constitutional isomers: 4-CMC, 3-CMC and 2-CMC. 4-CMC (4-CMC, 1-(4-chlorophenyl)-2-(methylamino)propan-1-one), also known as clefedrone, was first identified on the European drug market in June 2014. 4-CMC is still regularly found on the drug market in Europe, although it is now under the international control. 3-CMC (1-(3-chlorophenyl)-2-(methylamino)propan-1-one) was first identified on the European drug market in September 2014, based on a police confiscation in Sweden. In the case of 2-CMC (1-(2-chlorophenyl)-2-(methylamino)propan-1-one), although some reports indicate that it has been available on the drug market in at least two European countries since 2016, and its small quantities were also confiscated in 2016, the substance has not been formally reported to the EMCDDA (4).

The differentiation of these aforementioned three isomers requires the application of appropriate analytical techniques. Due to the variability in the reporting practices in Europe, the differentiation of CMC constitutional isomers is not performed in all forensic and toxicology laboratories. For this reason, some findings reported as one constitutional isomer may in fact be a different one (4).

The current knowledge of SC concentrations in blood samples is based mainly on the published case reports of intoxications or fatalities caused by SC intake. Very often, these substances are highly unstable, which makes proper interpretation of the determined concentrations challenging. It is also important to monitor a biomarker of CMC ingestion, namely, the dihydro-CMC metabolite, which shows extremely high stability under various storage conditions (5).

The purpose of the present study was to present and interpret the obtained toxicological analysis results of these cases, in which it was possible to determine or detect the presence of one of the isomers of CMC along with its intake biomarker—dihydro-CMC. These cases include deaths reported at the Department of Forensic Medicine in Kraków in 2016–2022.

## Experimental

### Analytical methods

The routine analysis involved the use of immunoassay, high-performance liquid chromatography (HPLC) coupled to diode array detection method in the Merck Tox Screening System developed by Merck (Germany), HPLC coupled to tandem mass spectrometry and gas chromatography coupled to mass spectrometry. The methods used have been published elsewhere (5–7). The blood samples were preserved in glass tubes with sodium fluoride and potassium oxalate. The urine samples were collected into glass tubes without preservatives.

### Casework

The analysis was undertaken as part of routine case investigations on behalf of the prosecutor’s office or the police using postmortem samples. Details of the cases are given in Table I. Drug testing involved an untargeted approach for common drugs of abuse, as well as covering alkaline, neutral and acidic medications. Ethyl alcohol was also tested, and carboxyhemoglobin (COHb) determination was performed when warranted.

**Table I.** Fatal Cases in Which It Was Possible to Determine or Detect the Presence of One of the Isomers of CMC along with Its Intake Biomarker—Dihydro-CMC

No.	Sex	Age	Case history	Time between the autopsy and toxicological analysis of biological material (months)	CMC concentration found in blood (ng/mL)	CMC found in urine	Others: NPS, drugs^a^ and alcohol determined in blood
1	F	20	Drug user, dependence from drugs, depression	3	4-CMC—50 Dihydro-4-CMC (+)	Vitreous humor: 4-CMC (+) Dihydro-4-CMC (+)	0.0‰ of ethyl alcohol
2	M	16	Drug user, mental disorder	3	4-CMC—20 Dihydro-4-CMC (+)	4-CMC (+) Dihydro-4-CMC (+)	U-47700 (854), *N*-desmethyl-U-47700 (342), *N,N*-didesmethyl-U-47700 (418), hydroxyzine (20), cetirizine (60) 0.0‰ of ethyl alcohol
3	F	29	Found dead after returning from birthday party; bright yellow substance scattered in bed sheets	2	3-CMC—95 Dihydro-3-CMC (+)		α-PVT (230), PV8 (40), PV-7 (6,400) 0.0‰ of ethyl alcohol
4	M	44	Drug and alcohol user, body revealed on stairs under the store	3	3-CMC—10 Dihydro-3-CMC (+)	3-CMC (+) Dihydro-3-CMC (+)	1.9‰ of ethyl alcohol
5	F	22	Drowning	5	3-CMC—36 Dihydro-3-CMC (+)	3-CMC (+) Dihydro-3-CMC (+)	Paracetamol (1,000) 1.4‰ of ethyl alcohol
6	M	24	Mental disorder. He fell out of a window on the second floor	2	3-CMC—< LOQ Dihydro-3-CMC (+)		Thiopental (2,300)^b^, pentobarbital (2,700)^b^, oxycodone (110)^b^, paracetamol (700)^b^, 4-MAA (30,800)^b^, 4-AA (5,700)^b^, 4-FAA (2,900)^b^ 0.0‰ of ethyl alcohol
7	M	33	He felt sick after smoking a “cigarette”. A string bag containing dried plant matter and a white substance was found	3	4-CMC—32 Dihydro-4-CMC (+)	4-CMC (+) Dihydro-4-CMC (+)	Metoprolol (31), ibuprofen (1,200), hydrocortisone (3,400) 0.0‰ of ethyl alcohol
8	M	29	Sudden cardiac arrest, drug addict, supposedly have taken a large amount of drugs and narcotics and sipped them with alcohol, human immunodeficiency virus (HIV) positive	5	4-CMC—15 Dihydro-4-CMC (+)	4-CMC (+) Dihydro-4-CMC (+)	*N*-Ethylhexedrone (1,240), *N*-ethylpentylone (27), midazolam (26)^b^, estazolam (30) 0.0‰ of ethyl alcohol
9	M	26	Found with no signs of life	4	4-CMC (−) Dihydro-4-CMC (+)	4-CMC (+) Dihydro-4-CMC (+)	Morphine (580), M3G (4,100), M6G (1,100) 0.0‰ of ethyl alcohol
10	M	23	He was found in his room with no signs of life. He was addicted to psychotropic drugs	4	4-CMC (−) Dihydro-4-CMC (+)	4-CMC (+) Dihydro-4-CMC (+)	MDMA (520), MDA (60), amphetamine (10), morphine (240), M3G (990), M6G (160), tramadol (680), lorazepam (16), diazepam (18), nordiazepam (11) 0.0‰ of ethyl alcohol
11	M	48	During a social gathering, the man was said to have taken a mephedrone solution	1	3-CMC—2,800 Dihydro-3-CMC (+)	3-CMC (+) Dihydro-3-CMC (+)	0.0‰ of ethyl alcohol
12	M	23	During a birthday party, he was stabbed in the subscapular area on the left side (under the heart). Cardiac arrest	3	3-CMC (−) Dihydro-3-CMC (+)	3-CMC (+) Dihydro-3-CMC (+)	Lidocaine (80) 2.6‰ of ethyl alcohol
13	M	22	He died in a fire resulted from setting the building on fire with a flammable substance and cutting off escape routes	1	4-CMC—38 Dihydro-4-CMC (+)	4-CMC (+) Dihydro-4-CMC (+)	1.1‰ of ethyl alcohol 35% of COHb
14	M	42	Found in a church apartment with a peripheral intravenous cannula (Venflon) inserted near his ankle	0 (1 day after the autopsy)	3-CMC—830 Dihydro-3-CMC (+)	3-CMC (+) Dihydro-3-CMC (+)	0.0‰ of ethyl alcohol
15	M	23	Found dead in a friend’s apartment. Cardiac arrest, death before arrival of the paramedics	5	3-CMC (−) Dihydro-3-CMC (+)	3-CMC (+) Dihydro-3-CMC (+)	MDA (19,700), MDMA (20), amphetamine (6) 0.5‰ of ethyl alcohol
16	M	25	Corpse revealed near the railroad embankment and the river	5	3-CMC (−) Dihydro-3-CMC (+)	3-CMC (+) Dihydro-3-CMC (+)	Cocaine (9), benzoylecgonine (470), ecgonine methyl ester (65) 0.0‰ of ethyl alcohol
17	M	26	Hanging/falling from a height, hands tied with a cord. Died after attempted hanging, after more than an hour of cardiopulmonary resuscitation (CPR). Treated psychiatrically, neuralgia, was addicted to drugs, took illegal substances	5	3-CMC (−) Dihydro-3-CMC (+)	3-CMC (+) Dihydro-3-CMC (+)	MDMA (950), MDA (130), buprenorphine (1.5), clonazepam (15), 7-AC (316), lamotrigine (1,100), amiodarone (3,100), mirtazapine (43), atropine (11) 0.0‰ of ethyl alcohol
18	M	44	Found in a hotel room. Hanging	4	3-CMC (−) Dihydro-3-CMC (+)	3-CMC (+) Dihydro-3-CMC (+)	Sildenafil (8) 0.0‰ of ethyl alcohol
19	M	17	Found behind the garages, near the school. A “bong” for smoking marijuana was found with him	2	4-CMC (−) Dihydro-4-CMC (+)	4-CMC (−) Dihydro-4-CMC (+)	ADB-BUTINACA (16), Δ^9^-THC (0.8), THC-COOH (6), THC-OH (1.8), (es)citalopram (190), *N*-desmethyl(es)citalopram (90), amphetamine (23), valproic acid (16,000) 0.0‰ of ethyl alcohol
20	M	27	Found in a car with no signs of life	3	4-CMC—100 Dihydro-4-CMC (+)	4-CMC (+) Dihydro-4-CMC (+)	0.0‰ of ethyl alcohol
21	M	29	Motorcyclist, traffic accident	7	3-CMC (−) Dihydro-3-CMC (+)	3-CMC (+) Dihydro-3-CMC (+)	0.0‰ of ethyl alcohol
22	M	27	Alcohol abuser. He was found dead in bed. He suffered from diabetes and had recently complained of abdominal and heart pain	8	4-CMC (−) Dihydro-4-CMC (+)	4-CMC (+) Dihydro-4-CMC (+)	Ketoprofen (60) 0.0‰ of ethyl alcohol
23	M	24	He was found dead in his apartment. He had previously been in drug rehabilitation treatment for drug addiction	6	4-CMC (−) Dihydro-4-CMC (+)	4-CMC (+) Dihydro-4-CMC (+)	Baclofen (1,850), promethazine (135) 0.0‰ of ethyl alcohol
24	F	28	Found in her apartment with no signs of life. She was receiving psychiatric treatment	3	4-CMC (−) Dihydro-4-CMC (+)	4-CMC (+) Dihydro-4-CMC (+)	Lamotrigine (17,700), aripiprazole (140), sertraline (95), norsertraline (700), codeine (50), C6G (60) 0.0‰ of ethyl alcohol
25	M	22	Found in the apartment with no signs of life, addicted to drugs and alcohol. Alcohol bottles and medicine blisters found next to the corpses	4	4-CMC (−) Dihydro-4-CMC (+)	4-CMC (+) Dihydro-4-CMC (+)	Morphine (380), M3G (660), M6G (110), 7-AC (160), lorazepam (218), diazepam (160), nordiazepam (70), temazepam (8), oxazepam (5), alprazolam (<LOQ), zolpidem (300), cocaine (<LOQ), benzoylecgonine (117), ecgonine methyl ester (20), cocaethylene (<LOQ) 0.9‰ of ethyl alcohol
26	M	18	Corpse found on the passenger seat of stolen, completely burned bus truck	5	4-CMC (−) Dihydro-4-CMC (+)	4-CMC (+) Dihydro-4-CMC (+)	Estazolam (27) 0.7‰ of ethyl alcohol 50% of COHb
27	M	25	Found with no signs of life, a syringe was found next to the corpse and he was addicted to drugs. A few hours before the body was found, he had an argument with his mother	4	3-CMC (−) Dihydro-3-CMC (+)	3-CMC (+) Dihydro-3-CMC (+)	Alprazolam (5), tramadol (17) 0.0‰ of ethyl alcohol

a Concentrations are in the unit of ng/mL.

b Drug most likely administered during medical procedures.

Abbreviations: (−), the substance was unidentified in the tested biological material; (+), the substance was identified in the tested biological material; 4-AA, 4-aminoantipyrine; 7-AC, 7-aminoclonazepam; ADB-BUTINACA, *N*-[(2*S*)-1-amino-3,3-dimethyl-1-oxobutan-2-yl]-1-benzyl-1 *H*-indazole-3-carboxamide; C6G, codeine-6-glucuronide; 4-FAA, 4-formylaminoantipyrine; <LOQ, the substance was below the limit of quantification of the method; M6G, morphine-6-glucuronide; M3G, morphine-3-glucuronide; 4-MAA, 4-methylaminoantipyrine; MDA, 3,4-methylenedioxyamphetamine; α-PVT, α-pyrrolidinopentiothiophenone; PV8 (α-PHPP), α-pyrrolidinoheptaphenone; PV-7 (α-PHP), α-pyrrolidinohexiophenone; THC-OH, 11-hydroxy-Δ^9^-tetrahydrocannabinol.

## Results and discussion

The limit of quantification, calibration curves range, accuracy and precision for 2-, 3- and 4-CMC were 10 ng/mL, 10–2,500 ng/mL, −19.9 to 7.3% and 7–10% relative standard deviation, respectively. Table I presents a summary of all cases (*n* = 27), in which one of the isomers of CMC and/or its dihydro metabolite was present. Identification of the isomer was carried out in each case based on a procedure using the trimethylsilylation process described in the authors’ earlier works (5, 6). In cases where a urine sample was available (for Case No. 1, a vitreous humor sample), it was qualitatively analyzed each time because of the higher stability of CMC in urine samples (5, 8).

**Figure 1.**
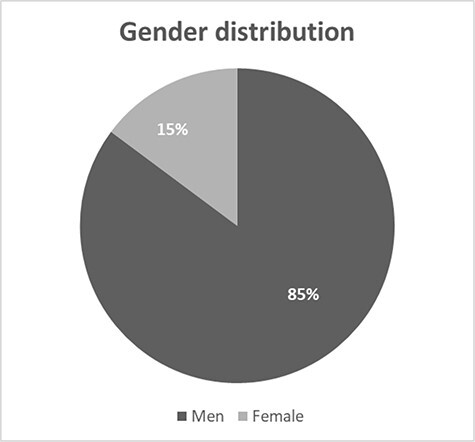
The gender distribution of CMC users.

**Figure 2.**
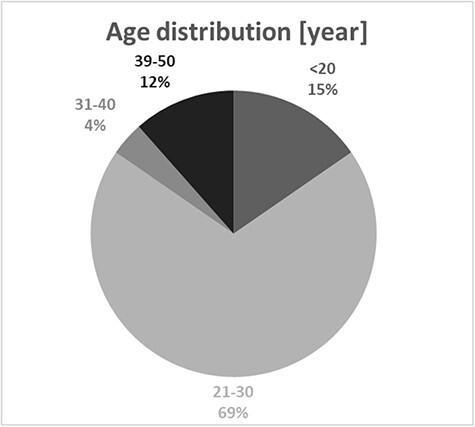
The distribution of CMC users by age.

**Figure 3.**
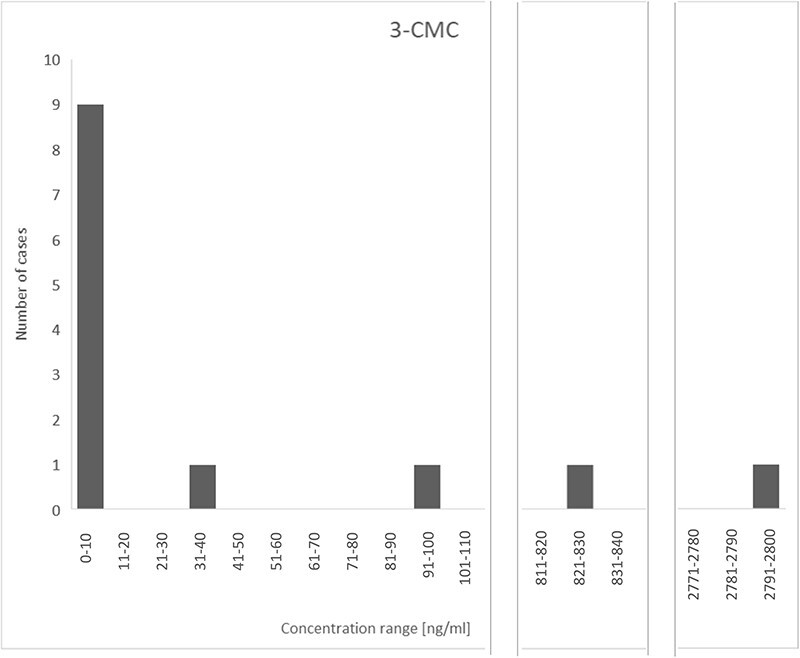
The concentration range of 3-CMC in the described cases.

**Figure 4.**
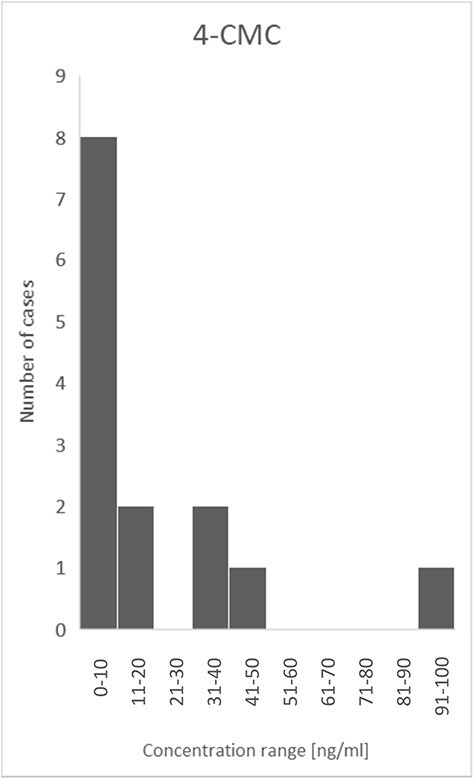
The concentration range of 4-CMC in the described cases.

In total, 85% (*n* = 23) of all presented cases were represented by men, and only 15% (*n* = 4) by women (Figure 1). The age range in the analyzed cases was quite wide, from 16 to 48 years. However, the average age was 27 years, and the median was 25 years (Figure 2). Thus, it can be seen that these substances are mostly taken by young men, perhaps more likely to experiment with NPS. Determined concentrations in blood samples for 3-CMC ranged from 10 to 2,800 ng/mL (Figure 3), and for 4-CMC, it ranged from 15 to 100 ng/mL (Figure 4). There was no evidence of 2-CMC or its dihydro metabolite in any of the presented cases.

**Figure 5.**
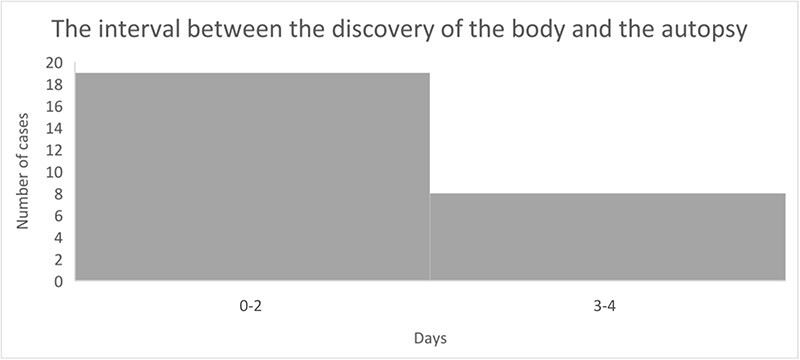
The interval between the discovery of the body and the autopsy.

An autopsy, depending on the case, was performed between 1 and 4 days after a body was found (Figure 5). The time between the autopsy and the toxicological analysis was determined by the ordering of toxicological tests by the prosecutor’s office (the average time was 4 months). Due to the circumstances of the case which were known, directing the toxicological investigation course for the presence of CMCs, the toxicological analysis was performed urgently in two cases, No. 11 and 14.

There was no correlation between the determined CMC concentrations and the time elapsed between autopsy and testing, which may be a result of, among other things, the amount of parent substance dose that was taken by the user, the variability of the time interval between death and disclosure of the body or the time of performing the autopsy examination itself.

In only 12 cases (44%), it was possible to detect and/or determine the parent substance, 3- or 4-CMC, in blood samples. Thus, in many cases, analysis of the blood sample alone for one of the CMC isomers would have yielded a false-negative result. Only monitoring the dihydro metabolite as a biomarker of CMC intake (5) and additional analysis of the urine sample can confirm CMC intake. Since we monitor dihydro metabolite in the Toxicology Laboratory of the Department of Forensic Medicine in Kraków, the number of positive results for one of the CMC isomers has increased significantly. This is because very often toxicology tests are ordered only for the blood sample, without analysis of the urine sample or other body fluids and organs.

In the case labeled No. 19, it was already possible to detect only the dihydro metabolite itself, even in the urine sample. On the other hand, relatively high levels of CMC concentrations were obtained in cases No. 11 and 14. In the case marked No. 11, in which the man was supposed to have consumed a mephedrone solution, the information obtained at an early stage about the circumstances of the incident made it possible to carry out the determination fairly quickly and obtain a high concentration of 3-CMC in the blood sample (2,800 ng/mL). However, this determination was performed 42 days after the autopsy, so the initial concentration was most likely much higher. In addition, a repeat extraction of the man’s blood sample after 2 months, when a decision was obtained that toxicological testing was necessary, showed an 18-fold decrease in the intensity of the analytical signal. In the case of No. 14 (3-CMC—830 ng/mL), the analysis was already possible the day after the autopsy, thanks to the preliminary results of the analysis of the fluid from the Venflon secured during the autopsy (5). On the other hand, in Case No. 20, a man who was found with no signs of life in the car, despite the fact that the test was performed 3 months after the autopsy, the parent substance was quantified at 100 ng/mL. The initial concentration could therefore have been much higher. In these men (Case Nos 11, 14 and 20) and in Cases Nos 1, 7, 22, 24 and 27, CMC poisoning was assumed as the cause of death (*n* = 8).

In cases of NPS intake, an in-depth risk assessment is mandatory and, as a general principle, the opinion of the unpredictability of the effects is taken into account. Also, since most cases involve the presence of several co-administered substances, and the parent substance is very often no longer detected in blood samples, interpretation of the analysis results obtained in terms of CMC toxicity is difficult. This is because in most of the cases discussed (*n* = 22), CMC is found in combination with other NPS (SCs, synthetic cannabinoids and synthetic opioids), classical drugs (amphetamines, opiates, cocaine and Δ^9^-tetrahydrocannabinol (Δ^9^-THC)), medications or ethanol. In two cases, COHb was additionally determined due to the circumstances of the incident—a fire (Case Nos 13 and 26). The cause of death in 20 cases (74%) was assumed to be xenobiotic poisoning. Of these, 12 cases involve the phenomenon of polytoxicomania.

In several cases (*n* = 7), the substances determined in the biological material were not the direct cause of death. In the Case No. 13, the man was trapped in a building, which was subsequently set on fire with the cutting off escape routes. The death was caused in the fire course as a result of high temperature/flame with carbon monoxide (CO) inhalation attached. In Case No. 26, the corpse of a man was found in a burned bus car, where the fire was most likely accidentally ignited under the influence of the drugs. The death occurred as a result of CO inhalation in the course of the fire. In Case No. 12, the man died as a consequence of his injuries, caused by a stab wound to the chest. In Case No. 18, the man’s cause of death was violent strangulation by hanging, and in the case of the woman marked No. 5, it was drowning. The cause of death of the man in Case No. 6 was cranio-cerebral injuries, resulting from a fall from a height. In the Case No. 21, the man’s death was the result of multiple organ injuries with destruction of the structures of the cranio-cervical junction and disruption of the brainstem, resulting from a traffic accident of a motorcyclist under the influence of 3-CMC.

Interesting in terms of medicolegal opinion seems to be Case No. 17, in which a man attempted to commit suicide by hanging himself. As a result of a rope breaking, the man fell from a height. The man was resuscitated, as a result of which a temporary return of circulation was achieved, which could indicate that the pressure on the neck alone was not yet sufficient to cause the man’s death as a result of the ischemia and thus hypoxia of the brain. The toxic effects of the substances in the man’s body, by possibly joining hypoxia of the central nervous system in the course of the attempted hanging, were considered decisive for the man’s death.

In the literature, mainly reported concentrations of 4-CMC are found and they are within a very wide range of concentrations. In blood samples collected in non-fatal cases, 4-CMC concentrations are found in the range of 0.181–75.3 ng/mL. In postmortem blood samples, on the other hand, concentrations in the range of 0.6–8,542 ng/mL were encountered (9–19). The concentrations of 4-CMC determined in the cases we presented are therefore within the range encountered in the literature for postmortem blood samples. In the case of 3-CMC, the only results published to date for the determination of 3-CMC in biological material are from the authors' earlier work. In the autopsy blood sample, the concentration of 3-CMC was 830 ng/ml (case No. 14 in this paper) (5). The limited number of reported cases of 3-CMC poisoning may be related to differences in reporting practices in Europe and the fact that differentiation of constitutional isomers of CMC is done in many, but not all, forensic and toxicology laboratories. Consequently, some of the detections reported as one constitutional isomer may in fact be a different one (4). In the cases we reported, 52% (*n* = 14) was 4-CMC and 48% (*n* = 13) was 3-CMC.

Published results may not reflect the actual concentration of CMC in the blood samples due to its low stability in the biological material. As Adamowicz and Malczyk (8) showed, the half-life of 4-CMC in a blank blood sample enriched with 4-CMC during storage at 5°C was 1 day, and the total decay time was about 4 months. In the case of 3-CMC, stability studies conducted on the authentic biological material showed complete decomposition of the parent substance in the blood sample after repeated extraction performed 2 months of storage at 4°C. On the other hand, storing an acidified blood sample under the same conditions allowed the detection of the parent substance in the blood sample for even 4 months (5). Thus, very often, we may have false-negative results for the presence of CMCs, since the parent substance may already be absent in the biological material at the time of toxicological testing.

Based on a serum sample from an authentic case of a 27-year-old man who was serving a prison sentence, Nowak et al. (9) obtained an initial 4-CMC determination of 11.5 ng/mL, which dropped to 4.0 ng/mL after 3 days of storing the sample at 4°C.

Another paper by the same authors (10) investigated the stability of 4-CMC in blood and vitreous humor samples. The study was based on samples taken during the autopsy of a 31-year-old man who was hospitalized after an alleged fall on a concrete floor. There was a 63% decrease in concentration in the blood sample after 30 days of storage at 4°C. The man had 66.2 ng/mL of 4-CMC in the blood sample and 181.5 ng/mL in the vitreous humor sample on the first day of analysis. In addition, 147.3 ng/mL amphetamine and 8.9 ng/mL atropine were determined in the blood sample, and 260.4 ng/mL amphetamine and 3.9 ng/mL atropine in the vitreous humor sample, respectively.

In most of the published cases in which CMC was present, as in the cases we presented, CMC most often occurs in the presence of other NPSs, classic drugs, medications or ethanol. Thus, very often, we are dealing with the earlier-mentioned phenomenon of polytoxicomania.

The paper by Tomczak et al. (11) showed the results of 4-CMC determination in 15 cases. The cases included non-fatal poisonings (*n* = 9), including driving under the influence, and fatalities (*n* = 6), including overdoses, suicides and traffic accidents. In only one case, 4-CMC appeared to be the only determined substance in the blood sample. The remaining cases showed the presence of other drugs, medications or ethyl alcohol. In the non-fatal cases, 4-CMC was present in the range of 1.3–75.3 ng/mL. In autopsy blood samples, the range was 56.2–1,870 ng/mL. Fourteen cases were men, with only one case of the woman. The ages of the individuals in the described cases ranged from 18 to 38 years.

In a study of SC poisoning cases in 2013–2019, Pieprzyca et al. (12) reported a range of 4-CMC concentrations in blood samples for eight fatal cases (seven men and one woman, aged 20–45 years), which ranged from 6 to 242 ng/mL, and for two non-fatal cases (a man aged 21 years and a woman aged 19 years), which ranged from 13 to 23 ng/mL.

The paper by Woźniak et al. (13) presented the results of 4-CMC determination in the two cases of biological material analysis collected during the autopsy. In both cases, in addition to 4-CMC, other substances from the NPS group and other drugs/medications were identified or determined. The concentrations obtained were 2.51 ± 0.19 ng/mL for the case of a 23-year-old female and 45.0 ± 1.3 ng/mL for the case of a 19-year-old female, respectively.

Zawadzki et al. (14) described the case of a 30-year-old man who was found naked in a state of decomposition in a car. They detected 4-CMC in the biological material at concentrations of 0.6 and 14.3 ng/mL in blood and urine samples, respectively. *N*-Ethylpentylone concentrations of 10.6 μg/mL in the blood sample and 17.6 μg/mL in the urine sample were also obtained. *N*-Ethylpentylone poisoning was considered the cause of death. However, the authors pointed out that the body was revealed in a state of putrefaction, which made it difficult to truly estimate *N*-ethylpentylone concentrations, as well as indicating possible degradation of 4-CMC in the biological material.

The work of Wiergowski et al. (15) described cases involving acute intoxication involving three men. A 23-year-old man was found dead after he jumped from the fifth floor of an apartment building (Male 2). There were two other aggressive and highly agitated men in the apartment, aged 23 years (Male 1) and 24 years (Male 3). One of the men (Male 1) died on the way to the hospital. Male 1 showed the presence in the blood sample of 4-CMC at a concentration of 2.14 ng/mL, 25B-NBOMe at a concentration of 66.5 ng/mL and 11-nor-9-carboxy-Δ^9^-THC (THC-COOH) at a concentration of 11 ng/mL. In the case of Male 2, 4-CMC was found in the blood sample at a concentration of 0.887 ng/mL and 25B-NBOMe at a concentration of 661 ng/mL. While in the case of Male 3, 4-CMC in the blood sample at 0:30 a.m. was at a level of 0.181 ng/mL, and at 9:30, it was already below the detection limit. In addition, 25B-NBOMe was determined at 34.8 ng/mL at 0:30 a.m. and 17.5 ng/mL at 9:30 a.m. The THC-COOH concentration of 13 ng/mL was also determined in the blood sample.

A case of a 24-year-old woman found dead in bed was reported in the study by Nowak et al. (16). The woman was supposedly addicted to NPS and had made several suicide attempts in the past. Five string bags containing white powder were revealed at the scene. Analysis of the woman’s blood samples revealed the presence of 1.7 ng/mL 4-CMC, 1,470 ng/mL 3,4-dichloro-*N*-[(1*R*,2*R*)-2-(dimethylamino)cyclohexyl]-*N*-methylbenzamide (U-47700), 89.5 ng/mL sertraline, 58.1 ng/mL *N*-ethylhexedrone, 18.0 ng/mL adinazolam and 8 ng/mL 4-chloro-*N*-isopropylcathinone (4-CIC). In the urine sample, 417 ng/mL 4-CMC, 3,940 ng/mL U-47700, 32.5 ng/mL sertraline, 147 ng/mL *N-*ethylhexedrone, 82.1 ng/mL adinazolam and 130 ng/mL 4-CIC were determined. The urine sample showed a significantly higher concentration of 4-CMC, which may be related to the increased stability of SCs in the urine sample. Toxicological interpretation of the results indicated fatal opioid U-47700 poisoning, in combination with other substances: SC, benzodiazepines and selective serotonin reuptake inhibitors.

Adamowicz et al. (17) described the case of an 18-year-old man who was found dead in his apartment. The man was supposed to have taken drugs regularly, including NPS. No 4-CMC was detected in the blood sample, while 1,477 ng/mL was determined in the urine sample. The blood sample showed 69 ng/mL alpha-pyrrolidinoisohexanophenone (α-PiHP), which was considered the direct cause of death. In addition, 1,351 ng/mL *N*-ethylhexedrone and 30 ng/mL benzoylecgonine were detected in the urine sample.

A study by Pieprzyca et al. (18) determined 4-CMC in two cases of suicide by hanging. In the first case of a 23-year-old man, 4-CMC was determined in the blood sample at a concentration of 381 ng/mL and in the urine sample at 632 ng/mL (THC-COOH: 15.1 ng/mL in the blood sample, 24.8 ng/mL in the urine sample; quetiapine: 70 ng/mL in the blood sample, 170 ng/mL in the urine sample; ethyl alcohol: 0.5‰ in the blood sample). The second case of a 19-year-old man showed only 4-CMC at 112 ng/mL in the blood sample and 499 ng/mL in the urine sample.

The work of Tusiewicz et al. (19) described the highest concentration of 4-CMC to the present moment. A 22-year-old man was taken to the emergency room (ER) from a party, where, according to a witness’ statement: “1.5 hours before he had ingested orally 20 grams of mephedrone”. The man showed impaired consciousness without logical verbal contact and had dilated pupils. His body temperature was 36.5°C, pulse was 100 beats per minute and breathing rate was 17 breaths per minute. During the diagnostic procedure, the condition of the patient rapidly deteriorated, which led to seizures and sudden cardiac arrest outbreak. Cardiopulmonary resuscitation was performed, but the patient was pronounced dead an hour after admission to the ER. In the biological material collected during the man’s autopsy, the determined concentrations of 4-CMC were 8,542 and 9,874 ng/mL in the blood sample and vitreous humor sample, respectively. Other substances present in both biological materials included drugs that were administered during hospitalization (atropine, diazepam, lidocaine and its metabolite norlidocaine) as well as exogenous compounds (methcathinone, ethyl alcohol). Due to the low stability of 4-CMC, the initial concentrations may have been even higher due to the time elapsed between death and toxicological analysis. This is because the material was stored at 4°C for 20 days before being analyzed.

In the published cases of poisonings or deaths involving CMC, men also predominated, and the age of the users was in the range of 18–45 years (9–19). These data therefore correspond to the cases presented in this paper. It is worth mentioning that most of the published cases come from Poland. This may be explained by the rather high popularity of SCs in Poland among users, including CMC.

SCs are popular and steadily taken by users due to expected effects like psychomotor stimulation, increased energy, increased ability to focus attention, euphoria, increased empathy, talkativeness, increased self-confidence and increased social interaction desire (20). However, considering that the composition of SC-containing products can vary significantly from package to package, even within the same batch, the effects of intake can be unpredictable. As published in the work of Grifell et al. (21), only 23.4% of samples contained the substance expected by the user. Samples that were expected to have 4-CMC contained both 4-CMC and 4‐bromomethcathinone (4-BMC). In contrast, 4-CMC was also detected in samples supplied as 3-methylmethcathinone (3-MMC), 3-CMC, ketamine or MDMA. Thus, a user may sometimes unknowingly intake NPS thinking they have purchased and are taking MDMA or ketamine.

The catalog of adverse side effects observed in SC users is wide and includes, on the cardiovascular side, symptoms such as tachycardia, hypertension, myocarditis, chest pain and cardiac arrest. However, the side effects of SCs do not only affect the cardiovascular system. On the nervous system side, symptoms such as concentration disorders, anxiety, depression, suicidal thoughts and attempts, psychosis, visual and auditory hallucinations, delusions, paranoia, aggressive behavior, seizures, headaches and dizziness, fainting and short-term memory impairment may occur. SC abuse can also result in mental and physical dependence. The following may also occur: abdominal pain, nausea, vomiting, liver damage, muscle and joint pain, skeletal muscle damage, paresthesias, kidney failure, electrolyte disturbances, accelerated and shallow breathing, respiratory failure, nose bleeding, hypersensitivity to light, nystagmus, excessive sweating, fever, insomnia and nightmares, among others (20).

## Conclusion

SCs constitute a major toxicological opinion challenge, in terms of toxicological evaluation of poisonings. As presented in this paper, a significant problem is their stability in the biological material and practices in the reporting of the obtained data. SCs, including CMC isomers, can often be undetected in biological material. This results in false-negative findings for CMC in toxicological opinions. Due to the low stability of SCs, it is important to monitor potential intake biomarkers that may show increased stability in the biological material. In the case of CMC isomers, the good biomarker of intake is the dihydro-CMC metabolite, which was detected in the blood sample in every case presented, even with the absence of the parent substance. The reported concentrations, on the other hand, do not reflect the actual concentrations of CMC at the time of the incident or death. Consequently, interpretation of the results obtained for CMC isomers in terms of assessing their toxicity and possible cause of death is difficult. However, it should be taken into account that in cases of NPS poisoning, an in-depth risk assessment is mandatory and the opinion of the unpredictability of the effects is taken as a principle.

## Data Availability

The data underlying this article are available in the article.
